# A poly(vinyl alcohol)/sodium alginate blend monolith with nanoscale porous structure

**DOI:** 10.1186/1556-276X-8-411

**Published:** 2013-10-04

**Authors:** Xiaoxia Sun, Hiroshi Uyama

**Affiliations:** 1 Department of Applied Chemistry, Graduate School of Engineering, Osaka University, Suita, Osaka 565-0871, Japan

**Keywords:** Monolith, Porous material, Poly(vinyl alcohol), Sodium alginate, Phase separation

## Abstract

A stimuli-responsive poly(vinyl alcohol) (PVA)/sodium alginate (SA) blend monolith with nanoscale porous (mesoporous) structure is successfully fabricated by thermally impacted non-solvent induced phase separation (TINIPS) method. The PVA/SA blend monolith with different SA contents is conveniently fabricated in an aqueous methanol without any templates. The solvent suitable for the fabrication of the present blend monolith by TINIPS is different with that of the PVA monolith. The nanostructural control of the blend monolith is readily achieved by optimizing the fabrication conditions. Brunauer Emmett Teller measurement shows that the obtained blend monolith has a large surface area. Pore size distribution plot for the blend monolith obtained by the non-local density functional theory method reveals the existence of the nanoscale porous structure. Fourier transform infrared analysis reveals the strong interactions between PVA and SA. The pH-responsive property of the blend monolith is investigated on the basis of swelling ratio in different pH solutions. The present blend monolith of biocompatible and biodegradable PVA and SA with nanoscale porous structure has large potential for applications in biomedical and environmental fields.

## Background

Polymer-based monoliths which emerged in the early 1990s have attracted significant attention during about 20 years of progress. Up to now, they have been applied for various fields such as chromatography, biomolecule immobilization, and support catalysis, because of their predominant pH stability, nonspecific interaction, and fast mass transfer performance [1-4]. However, their main drawbacks include the limit of small surface area for the pore walls and the lack of functional groups on the pore surface [5,6].

Stimuli-responsive porous materials have aroused special interest not only for their pore structures, but also because they can go through the visible changes in their property to respond to environmental variation [6]. Some efforts have been made to introduce functional groups onto the pore surface of polymer monoliths, providing stimuli-responsive properties [7]. In most cases, such monoliths should be fabricated by polymerization of monomers and subsequent surface functionalization. For both processes, time-consuming procedures for precise control of the monolith structure and introduction ratio of the functional group are often involved.

Recently, we developed a novel method for preparation of the polymer-based monolith directly from a polymer by means of either thermally induced phase separation or non-solvent induced phase separation (NIPS). This phase separation technique represents a very simple and straightforward approach to the formation of a monolith having a uniform nanoscale porous structure (mesoporosity) without assistance of any templates in comparison with conventional fabrication methods from monomers. In NIPS, the addition of non-solvent into a homogeneous polymer solution with appropriate ratio of solvent and non-solvent affords the monolith with a uniform pore structure. So far, we have fabricated monoliths of hydrophobic polymers such as polyacrylonitrile, polycarbonate, and polymethacrylates through this method [8,9].

Very recently, we reported fabrication of a poly(vinyl alcohol) (PVA) monolith via thermally impacted non-solvent induced phase separation (TINIPS) [10]. This method is based on NIPS and a thermal factor is moreover introduced. The PVA monolith bearing many hydroxyl groups possesses a large surface area and a uniform nanoscale porous structure; thus, the hydrophilic PVA monolith has a large potential for bio-related and environmental applications. In this study, the fabrication of a blend monolith of PVA and sodium alginate (SA) has been examined for further functionalization of the PVA monolith.

Although fabrication of monoliths consisting of more than two polymers is expected to broaden their applications in various fields, it is generally difficult to realize due to the different conditions of phase separation of the blended polymers. In many cases, only one polymer is forward subjected to the phase separation, in which others remain in the solution of the phase separation system. Previously, we successfully fabricated a blend monolith of polycarbonate and poly(3-hydroxybutyrate-co-3-hydroxyhexanoate) by precise choice of a solvent via NIPS, in which case, the solvent of the phase separation is the same as that for monolith fabrication of each polymer by NIPS [11].

SA is a kind of anionic polysaccharides having a carboxylate group in the side chain. It has excellent features such as biocompatibility, biodegradability and pH-responsive property. Based on these characteristics, SA is often used as matrix of biomaterials. The carboxylate group of SA is reported to form hydrogen bonding with the hydroxyl group of PVA [12,13]; however, there have been few literatures focusing on the phase separation in bulk fabricated by blending of PVA and SA. Furthermore, a monolith of SA has not been fabricated up to the present. This study deals with the facile fabrication of a PVA/SA blend monolith via TINIPS on the basis of this hydrogen bonding formation. A mixed solvent of methanol and water enables the fabrication of this blend monolith, whereas the PVA monolith is formed in an aqueous acetone. To our best knowledge, SA is incorporated in polymer monoliths by selection of appropriate phase separation conditions for the first time.

## Methods

### Materials

Sodium alginate powders and PVA powders with a hydrolysis ratio of 98% were purchased from Wako Pure Chemical Industries, Ltd (Tokyo, Japan). All other reagents and solvents were used as received.

### Preparation of PVA/SA blend monolith

An aqueous solution of a mixture of PVA and SA (95:5 wt.%) is prepared by dissolving these polymers into water at 95°C. After cooling the polymer solution to 60°C, methanol as non-solvent is added dropwise. Afterward, the mixture is kept at 20°C for 36 h, during which period the phase separation occurs to form the monolithic column. The monolith is then immersed into the calcium chloride solution for ionical cross-linking of SA. After 2 h, the monolith is washed repeatedly by acetone to remove the solvent and subsequently dried under vacuum.

### Characterizations

Scanning electron microscopic (SEM) images are recorded on a Hitachi S-3000N instrument (Tokyo, Japan) at 15 kV. The samples are cut with a scalpel and coated with a thin layer of gold using an ion sputter apparatus (E-1010 Ion Sputter, Hitachi Ltd, Tokyo, Japan). Nitrogen adsorption/desorption isotherms are measured with a NOVA 4200e surface area and pore size analyzer (Quantachrome Instruments, Boynton Beach, FL, USA) at 25°C. The Brunauer Emmett Teller (BET) method is utilized to determine specific surface areas. Before the measurements, all samples are degassed at 25°C for 12 h under vacuum. Fourier transform infrared (FT-IR) measurements by the attenuated total reflectance (ATR) method are performed using the Thermo Scientific (Yokohama, Japan) Nicolet iS5 with iD5 ATR accessory.

Porosity of the monolith samples is measured using a gravimetric method according to the following equation:

$$\text{Porosity}\left(\%\right)=\left(1-V_{1}/V_{0}\right)\times 100,$$

where *V*_*1*_ is the volume of a certain weight of the PVA/SA blend powder and *V*_0_ is the volume of the same weight of PVA/SA blend monolith.

The pH-sensitivity of PVA/SA blend monolith samples is evaluated on the basis of the swelling ratio in a solution with different pH, which is determined by the following equation [14]:

$$\text{Swelling}\;\text{ratio}\left(\%\right)=W_{b}/W_{e}\times 100,$$

where *W*_*e *_and *W*_*b *_are the weights before and after immersion, respectively.

## Results and discussion

**Figure 1:**
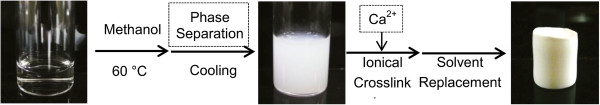
Fabrication process of PVA/SA blend monolith via TINIPS.

The general synthetic procedure is shown in Figure 1. For the fabrication process, selection of non-solvent and the ratio of solvent and non-solvent are crucial factors for the formation of the blend monolith. The detailed screening of the phase separation solvent shows that a mixture of water and methanol with a ratio of 2:3 is the most suitable. Intriguingly, the PVA monolith with good mechanical strength is not formed in this solvent. When the methanol ratio of the mixed solvent is more than 60%, the precipitation takes place very quickly during the phase separation, resulting in no formation of the monolith. On the other hand, no phase separation occurs when the methanol ratio is less than 60%. These behaviors can be rationalized as follows. After adding methanol into the polymer solution, the mixed solvent system transforms into polymer-rich phase and polymer-lean phase. As the amount of non-solvent (methanol) increases, the polymer segments in the polymer-rich phase become folded and aggregated, leading to the increase of the concentration in the polymer-rich phase. When the increasing concentration reaches to a certain degree, the phase separation takes place. In the case of a smaller amount of non-solvent, the concentration of polymer-rich phase is not high enough to induce the phase separation; while for a much larger amount of non-solvent, a mass of polymer segments aggregate rapidly, resulting in precipitation of the polymer in the phase separation system.

Moreover, the ionical cross-linking with Ca^2+^ is an essential process for the blend monolith fabrication. Without this step, the blend monolith turns out to be drastically shrunk in the drying process and the pore structure is not maintained any more. It is probably because the hydrogen bonds formed between PVA and SA are not strong enough to keep the porous structure of the blend monolith; the cross-linked structure of SA with Ca^2+^ enhances the strength of the blend monolith with preservation of the porous morphology [15].

**Figure 2:**
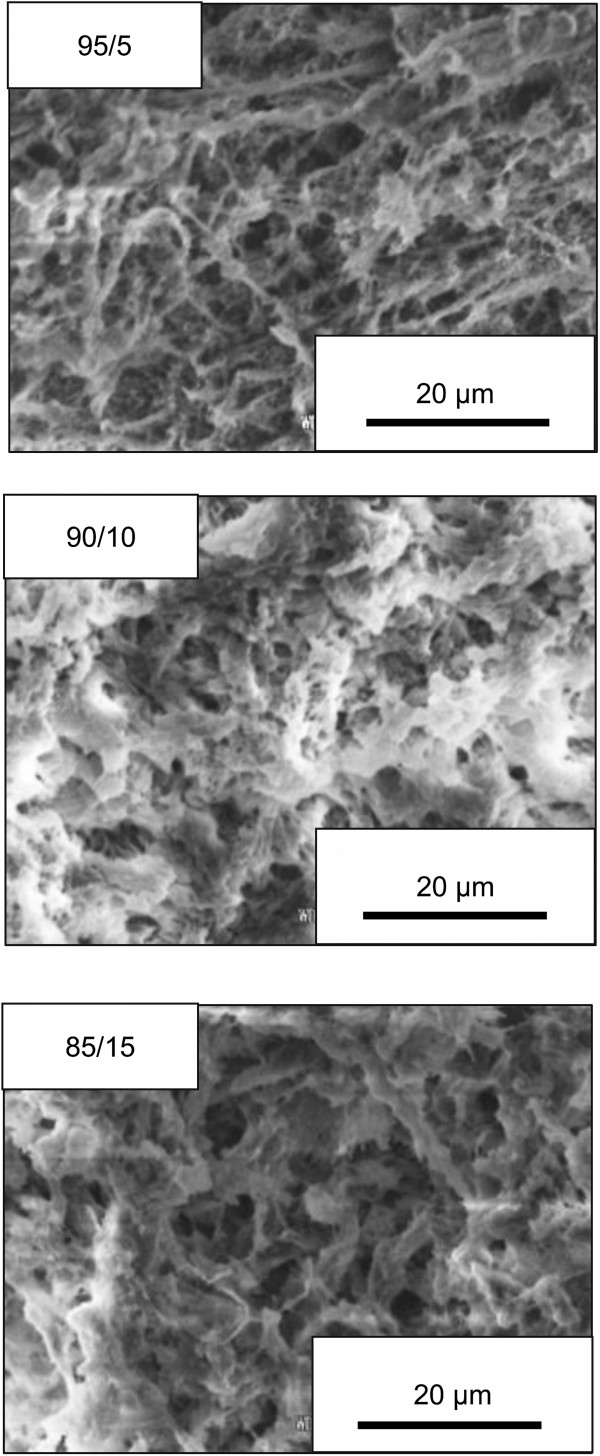
SEM images of PVA/SA blend monoliths with different SA contents.

The blend monoliths with different mixed ratios of PVA/SA = 95/5, 90/10, and 85/15 (PVA/SA-1, PVA/SA-2, and PVA/SA-3, respectively) are successfully fabricated under the conditions described above. The mixed ratio strongly affects the formation of the blend monolith. When the ratio of PVA/SA is 70/30, the monolith is not formed due to the very high viscosity of the solution, not suitable for the phase separation. Figure 2 shows the SEM images of the PVA/SA blend monolith with different mixed ratios of PVA/SA. Similar pore structures are observed in all the blend monoliths. In the case of low ratio of SA (5%), a continuous interconnected network is well formed. With increasing the content of SA, the skeleton size increases and the pore size decreases, which affect the interconnectivity of the pore structure. This behavior is explained as follows [16]. The viscosity of the solution increases with increasing the content of SA, which leads to the higher degree of entanglement and the slower dynamics of phase separation. Furthermore, the formation of the soluble complex between PVA and SA may also delay the phase separation process.

**Figure 3:**
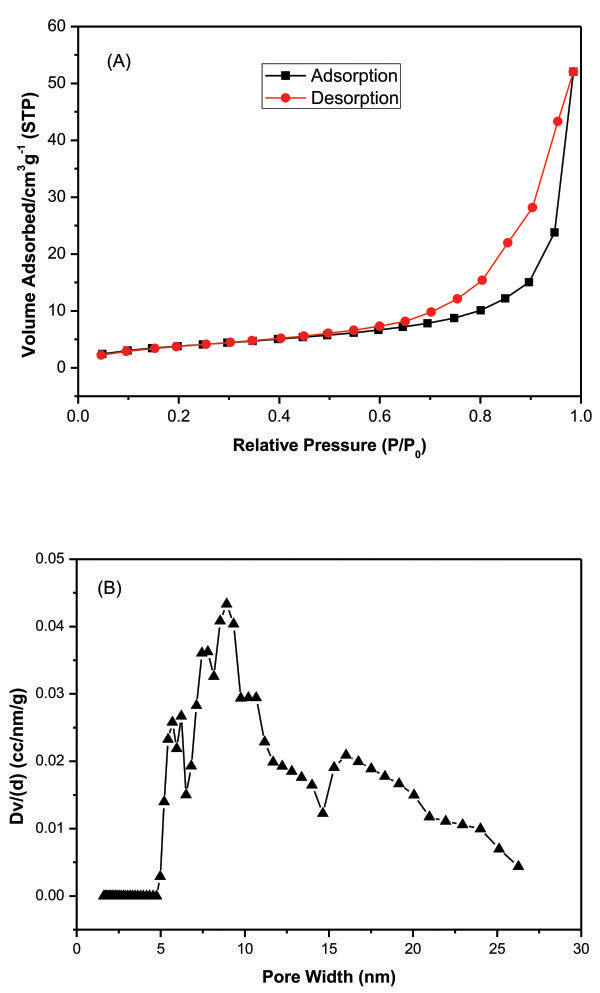
Nitrogen adsorption-desorption isotherms of PVA/SA blend monolith (PVA/SA-1) (A); pore size distribution by NLDFT method (B).

Nitrogen adsorption-desorption isotherm of the blend monolith (PVA/SA-1) is shown in Figure 3A. It belongs to a type II isotherm which is formed by a macroporous absorbent. The macroporous structure is confirmed by the SEM images (Figure 2). Besides, a type H3 hysteresis loop in the P/P_0_ range from 0.5 to 1.0 is observed. This hysteresis loop is caused by capillary condensation, suggesting the existence of more or less slit-like nanoscale porous structures in the present blend monolith [17]. The BET surface area of PVA/SA-1 is 89 m^2^/g, revealing the relatively large surface area of the obtained monolith. The pore size distribution (PSD) plot of the sample obtained by the non-local density functional theory (NLDFT) method is shown as Figure 3B. The PSD of the blend monolith is centered at 8.9 nm in the range from 5.0 to 26 nm. The data clearly confirms the nanoscale porous structure of the blend monolith.

The BET surface areas of PVA/SA-2 and PVA/SA-3 are 54 and 91 cm^2^/g, respectively, which are close to that of PVA/SA-1. The porosity values of PVA/SA-1, PVA/SA-2, and PVA/SA-3 calculated from the equation mentioned above are 85%, 84%, and 87%, respectively. These data indicate that the blend monolith obtained by TINIPS method possesses very high porosity, which may be important for the potential applications.

**Figure 4:**
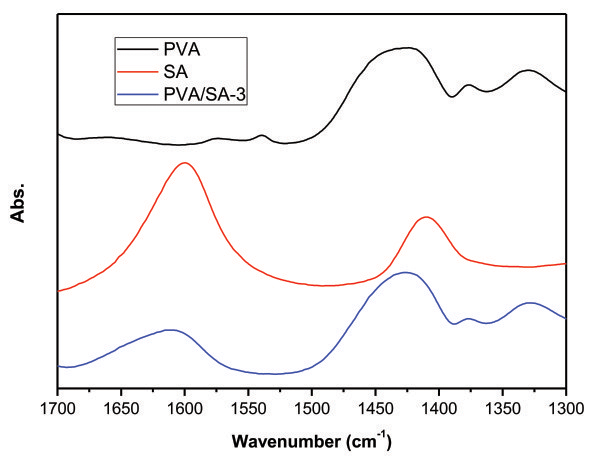
FT-IR spectra of PVA, SA, and the PVA/SA monolith (PVA/SA-3).

Figure 4 shows FT-IR spectra of PVA, SA, and the blend monolith (PVA/SA-3), which clearly implies that the blend monolith consists of both polymers. In the spectrum of SA, peaks at 1,600 and 1,410/cm are ascribed to asymmetric and symmetric carboxylate stretching vibrations of SA, respectively. These two vibrations are also observed in all the spectra of the blend monoliths and shift to a higher frequency range. These data clearly suggest the strong interaction between PVA and SA in the blend monolith [14]; the hydrogen bond between the carboxyl group of SA and hydroxyl group of PVA is formed. This interaction may be related to the specific solvent of the phase separation for the combination of PVA and SA.

**Figure 5:**
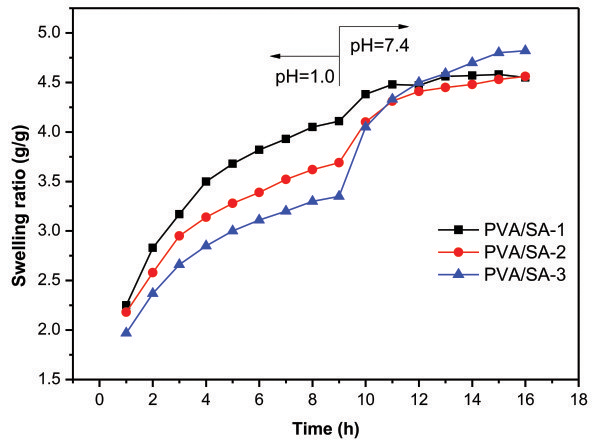
Effect of pH on swelling behaviors of PVA/SA blend monoliths.

The pH-sensitive property of the PVA/SA blend monolith with different mixed ratios is shown in Figure 5. At first, the dried blend monolith is placed in an acidic solution (pH 1.0). The monolith is gradually swollen. After 9 h, the sample is transferred into in a neutral solution (pH 7.4). Under the acidic condition, the swelling ratio decreases with increasing the SA content; while the swelling ratio significantly increases as the SA content increases under the neutral condition. This behavior can be explained by the acidic form of the carboxylate group of SA in pH 1.0 and the neutralized form in pH 7.4; the electrostatic repulsion of the carboxylate group increases, leading to the increase of the swelling ratio [18-20].

## Conclusions

The PVA/SA blend monolith with nanoscale porous structure and pH-responsive property is successfully fabricated via TINIPS without any templates. We have first achieved the fabrication of a monolith containing SA by the appropriate selection of the solvent for the phase separation.

PVA and SA are widely used as biomaterials due to their good biocompatibility. A combination of this feature and nanoscale structural characteristics of the present blend monolith offers promising prospects for the applications in bio-related and environmental fields. SA provides the pH-sensitive property in the blend monolith, which may be potentially useful for controlled drug delivery systems. Moreover, the present study is highly significant to suggest the possibility to fabricate blend monoliths consisting of bioactive polymers which can not form monolithic structure solely. Further studies on the fabrication of blend monoliths of functional polymers and their bio-related applications are under way in our laboratory.

## Competing interests

The authors declare that they have no competing interests.

## Authors' contributions

HU conceived and guided the experiment, and XS carried out the total experiment. Both authors participated in the analysis of data. XS drafted the manuscript. HU guided the revision of the manuscript. Both authors read and approved the final manuscript.
